# Contrasting a Mobile App With a Conversational Chatbot for Reducing Alcohol Consumption: Randomized Controlled Pilot Trial

**DOI:** 10.2196/33037

**Published:** 2022-05-16

**Authors:** Patrick Dulin, Robyn Mertz, Alexandra Edwards, Diane King

**Affiliations:** 1 Department of Psychology University of Alaska Anchorage Anchorage, AK United States; 2 Center for Behavior Research and Services University of Alaska Anchorage Anchorage, AK United States

**Keywords:** alcohol, hazardous drinking, smartphone app, chatbot, brief intervention, effectiveness, utilization, mobile phone

## Abstract

**Background:**

Mobile apps have shown considerable promise for reducing alcohol consumption among problem drinkers, but like many mobile health apps, they frequently report low utilization, which is an important limitation, as research suggests that effectiveness is related to higher utilization. Interactive chatbots have the ability to provide a conversational interface with users and may be more engaging and result in higher utilization and effectiveness, but there is limited research into this possibility.

**Objective:**

This study aimed to develop a chatbot alcohol intervention based on an empirically supported app (Step Away) for reducing drinking and to conduct a pilot trial of the 2 interventions. Included participants met the criteria for hazardous drinking and were interested in reducing alcohol consumption. The study assessed utilization patterns and alcohol outcomes across the 2 technology conditions, and a waitlist control group.

**Methods:**

Participants were recruited using Facebook advertisements. Those who met the criteria for hazardous consumption and expressed an interest in changing their drinking habits were randomly assigned to three conditions: the Step Away app, Step Away chatbot, and waitlist control condition. Participants were assessed on the web using the Alcohol Use Disorders Identification Test, Adapted for Use in the United States, Readiness to Change Questionnaire, Short Inventory of Problems-Revised, and Timeline Followback at baseline and at 12 weeks follow-up.

**Results:**

A total of 150 participants who completed the baseline and follow-up assessments were included in the final analysis. ANOVA results indicated that participants in the 3 conditions changed their drinking from baseline to follow-up, with large effect sizes noted (ie, *η*^2^=0.34 for change in drinks per day across conditions). However, the differences between groups were not significant across the alcohol outcome variables. The only significant difference between conditions was in the readiness to change variable, with the bot group showing the greatest improvement in readiness (*F*_2,147_=5.6; *P*=.004; *η*^2^=0.07). The results suggested that the app group used the app for a longer duration (mean 50.71, SD 49.02 days) than the bot group (mean 27.16, SD 30.54 days; *P*=.02). Use of the interventions was shown to predict reduced drinking in a multiple regression analysis (*β*=.25, 95% CI 0.00-0.01; *P*=.04).

**Conclusions:**

Results indicated that all groups in this study reduced their drinking considerably from baseline to the 12-week follow-up, but no differences were found in the alcohol outcome variables between the groups, possibly because of a combination of small sample size and methodological issues. The app group reported greater use and slightly higher usability scores than the bot group, but the bot group demonstrated improved readiness to change scores over the app group. The strengths and limitations of the app and bot interventions as well as directions for future research are discussed.

**Trial Registration:**

ClinicalTrials.gov NCT04447794; https://clinicaltrials.gov/ct2/show/NCT04447794

## Introduction

### Background

Access to evidence-based treatment and support for addressing excessive alcohol use is a public health priority given that alcohol continues to be the third leading preventable cause of death in the United States, and its excessive use is responsible for >95,000 deaths each year [1,2]. A recent review of death certificates found that the annual number of deaths from alcohol-related causes doubled between 1999 and 2017 for individuals aged ≥16 years [3]. Although the effectiveness of behavioral interventions for alcohol addiction is well established [4,5], the National Epidemiological Survey on Alcohol and Related Conditions found that treatment utilization is very low; in 2019, among the 14.5 million people aged ≥12 years reporting a past year alcohol use disorder, only 7.6% received treatment for alcohol use at any location [6].

### Technology-Delivered Alcohol Interventions and Health Equity

Technology-based interventions, including mobile apps, have great potential to meaningfully expand access to treatment and have been shown to be acceptable among alcohol and other substance users [7-9]. Over the past 15 years, numerous behavior-change interventions have been created to capitalize on the potential of the internet, including several alcohol interventions with demonstrated effectiveness in reducing alcohol consumption without the guidance of a counselor [10-13]. For example, the Drinker’s Checkup, a web-based brief motivational intervention that provides alcohol-use assessment, individualized feedback, and an intervention to develop a plan of behavior change, reduced alcohol consumption among problem drinkers by 50%, with reductions maintained at the 12-month follow-up [14]. A meta-analysis found that effect sizes from technology-based interventions were “as effective or nearly as effective as face-to-face therapy” [15], with clinical benefits found in other reviews [16,17]. In addition to their effectiveness, technology-based interventions have great potential to reduce health disparities in hidden populations [18,19], including homeless individuals [20]. Whether technology is used to deliver direct treatment services [19], or to provide behavioral support for reducing alcohol use or preventing relapse [21], technology-based alcohol interventions overcome several personal and access-related barriers to in-person alcohol treatment, including poor or inadequate availability of services; cost and inadequate insurance; convenience in the face of childcare, work, and transportation challenges; and beliefs about help-seeking as shameful or a sign of weakness [22,23]—barriers to treatment that are particularly salient for women, minorities, and those in rural locations [23-25]. Relatedly, technology-based interventions have the potential to address concerns about privacy and stigma that may be associated with attending alcohol treatment facilities [25,26].

### Mobile Devices and Contextual Relevance

One important advantage of technology-based alcohol interventions is that they can be accessed from home and the intervention is situated within daily contexts [27-29]. Much of what leads to continued alcohol use or relapse occurs in an individual’s everyday environment, where, unlike a traditional treatment context, exposure to alcohol-related cues, stress, and negative affect are uncontrolled [30,31]. These contextual and situational cues may overwhelm an individual’s coping resources and other skills that were discussed during treatment sessions with the hope that they would be used when facing cravings for alcohol triggered by internal and external factors encountered in daily life [31,32]. The increased use of smartphones to access the internet is especially promising, as these mobile devices provide a way to support behavior-change goals whenever and wherever needed. While digital divide issues persist with regard to high-speed internet access, it is estimated that 81% of all American adults, 79% aged 50 to 64 years and 53% aged ≥65 years, own smartphones [33]. These data also indicate that minority groups are at least as likely as White individuals to own a smartphone, and recent trends show a preference for smartphone use over desktop access to the internet [33], suggesting an opportunity to improve treatment access for historically underserved groups.

Similar to other internet-based interventions, smartphone-based interventions help bridge the gap between those in need of treatment and those receiving it by addressing stigma concerns associated with treatment program attendance, leveraging the desire to independently manage an alcohol problem, and eliminating the need for physical travel to a treatment facility. In addition, as smartphones are carried at almost all times by their owners, they have greater potential to provide timely interventions in the actual environment in which drinking occurs, and given that most mobile health apps are free or sell for <US $5, they are much more affordable than traditional treatment. That being said, the growing volume of publicly available apps varies in quality and effectiveness, and even those developed using theories of behavior change and evidence-based content may lack rigorous evaluation. In a recent systematic review of health behavior change apps, including diet, physical activity, and alcohol use, many reported improvements in targeted outcomes, but few demonstrated significant treatment effects over comparison groups in randomized trials [34]. The reported limitations included study design issues (eg, nonrepresentative samples and inadequate comparison groups), intervention design issues (eg, features offered are not based on theory or existing evidence), and limited or short-lived engagement with the app [30,34].

### Treatment Effectiveness and Effective Management

Maintained engagement with behavior change apps has been associated with app effectiveness in a number of studies and may depend on specific design features that improve adherence and efficacy. Engagement-enhancing features include visually appealing and easily navigated content; features based on behavior-change principles including feedback, self-monitoring, and data-driven adaptation; and features that promote therapeutic alliance including acceptance and support, relatability, and positive expectations [35]. For example, conversational agents that use artificial intelligence to proactively guide, prompt, and check in with participants and encourage general use of the app have been found to improve the therapeutic alliance between the participant and the intervention, resulting in more engagement [36]. In other studies, the ability to set goals, self-monitor progress, and receive feedback increased engagement across a variety of health behaviors, including alcohol use [37]. An advantage of apps is their ability to generate utilization data, which are commonly used as a proxy for quantifying engagement. Utilization metrics typically include the number of log-ins, proportion of features accessed, and frequency and duration of use [30]. However, interpreting the relative importance of these metrics to determine the amount and type of engagement that is *sufficient* to achieve the desired behavioral and health outcomes (ie, effective engagement) has not been established [37]. Given the lack of practical measures to comprehensively assess effective engagement, researchers continue to rely on utilization data with the assumption that more engagement is better [38].

### Step Away: A Behavior Change App for Reducing Excessive Alcohol Use

Step Away is a smartphone app designed to deliver empirically based alcohol assessment and intervention for individuals who drink at hazardous levels that may present health risks. Step Away is the next generation of an earlier app that we tested (the Location-Based Monitoring and Intervention System-Alcohol) with individuals with an alcohol use disorder, which demonstrated significant 6-week reductions in alcohol consumption, along with ratings by participants as being very helpful in changing their drinking habits [39]. This study also indicated that the amount of use of Location-Based Monitoring and Intervention System-Alcohol features was related to changes in alcohol consumption. The design of Step Away is informed by three theoretical constructs that are considered the key *active ingredients* for person-centered, behavioral-based intervention and the treatment of addictions: motivational enhancement [40], relapse prevention [41], and community reinforcement [42]. The app offers eight modules in addition to daily alcohol consumption and craving tracking: (1) *assessment and feedback* on alcohol consumption relative to age-specific norms, drinking-related problems, and monetary costs of drinking, including daily prompting to complete a brief questionnaire on drinking behavior and cravings during the prior 24 hours, and weekly feedback highlighting progress toward goals; (2) *goal setting*, which asks participants to select abstinence or moderation as a goal; (3) *rewards*, which prompts them to set up a reward for meeting their goal and reminds them to reward themselves when their goal is met (eg, 30 days of no drinking); (4) *cravings*, which offers 6 in-the-moment interventions for coping with cravings; (5) *moderation or abstinence strategies*, which consists of simple behavioral strategies tailored to the participant’s goal; (6) *supportive persons*, which provides tools for connecting with participant-identified friends or family when additional support is needed; (7) *reminders*, which encourages the creation of visual reminders of their reasons for changing their drinking habits, including the ability to upload inspirational photos to make a change; and (8) *new activities,* which recommends healthy behaviors and the ability to schedule selected activities within the smartphone calendar. Step Away also provides real-time intervention options; that is, when a participant clicks on the “Get Help” icon, they are provided with strategies for managing cravings or negative emotions and contacting a national treatment finder service to receive help finding in-person treatment in their area.

Although engagement with Step Away has been shown to be relatively high with a recent study of the app in a veteran sample showing that approximately 40% of participants were still engaged with the app at 6 months [43], use of the treatment *steps* or other modules was found to be relatively low [43]. In an attempt to increase intervention engagement, we developed a chatbot version of Step Away that incorporates the app modules into a chat-delivered intervention, which we postulated could result in higher use and engagement over the Step Away app. A chatbot is a computer program that simulates human conversation powered by pre-established rules and artificial intelligence. They have become common in e-commerce, call centers, and internet gaming. They serve as digital assistants that communicate with a user through texting to help a user with numerous tasks, such as planning air travel or helping with a bank transaction. In the smartphone context, they are being used in texting interfaces, in such a way that it appears to the user that they are having an actual conversation with the service. An important distinction between apps and bots is that a bot user has the perception of talking with the service. Bots are emerging as promising tools in contemporary health care [44]. An example of a health care chatbot is Melody, which has a text conversation with a patient about their symptoms and provides potential diagnoses that a health care practitioner can then use to develop a treatment plan [45].

A key difference between the app and bot is that the app relies on the user to launch a new feature on their own, whereas the bot guides the user through a conversational interface and essentially serves as an alcohol reduction personal assistant. The bot is also designed to perform a daily check-in regarding ongoing drinking and cravings and offers a menu of in-the-moment tools that can be used to manage the situation or experience.

### Objective

This paper presents results from a 3-month pilot study that compared effectiveness and participant engagement differences among individuals randomly assigned to one of three groups: (1) Step Away app, (2) Step Away bot, and (3) assessment-only delayed condition (control). Our study builds on the existing literature by indicating whether a chatbot-delivered version of a smartphone-delivered intervention has superior engagement and alcohol outcomes compared with an app with similar intervention features over a 12-week duration, and whether these interventions produce superior outcomes over a waitlist control condition.

## Methods

### Study Design

The study used a randomized controlled study design and a mixed methods approach (ClinicalTrials.gov NCT04447794). Participants were enrolled and randomly assigned to one of three groups: Step Away app (for iPhone and Android smartphones), Step Away chatbot, and assessment-only delay (control). Participants were assessed for their alcohol consumption and related behaviors when they enrolled in the study (baseline) and again 12 weeks later (follow-up). Age and gender stratification were used to ensure a relatively even distribution across each intervention or control group.

### Recruitment

Participant recruitment was conducted through Facebook advertisements, which provided a link to the study website and the web-based prescreening survey. Recruitment began in early June 2021 and was completed in early September 2021 when the target sample size was reached. Advertisements were targeted to all Facebook users who may meet the following criteria: age ≥18 years; have either an iPhone or an Android phone; reside in the United States; not in another form of alcohol treatment or using another mobile health alcohol intervention, be an active drinker, and have proficiency in English language.

The prescreening survey asked potential study participants about these criteria as well as all 10 questions from the Alcohol Use Disorders Identification Test, Adapted for Use in the United States (USAUDIT). Those who met these criteria, and who had a USAUDIT score between 8 and 24 inclusive for males aged ≤65 years and a score between 7 and 24 inclusive for females as well as males aged ≥65 years, were invited to complete the consent form and the baseline survey. The target audience for Step Away is those whose drinking patterns fall in the at-risk or high-risk USAUDIT zones. Therefore, those with USAUDIT scores of ≥8 for males and ≥7 for females met the criteria for risky drinking; those with scores of ≥25 were not eligible to participate. A US $25 Amazon e-gift card was provided to each participant when they completed the baseline survey, and another US $25 Amazon e-gift card was emailed after the completion of the follow-up survey.

### Measures

The following measures were assessed at baseline and follow-up. The USAUDIT was assessed at the time of screening for eligibility and again at baseline for comparison. Demographic characteristics such as age, sex, education, and ethnicity were collected.

#### Alcohol Use Disorder Identification Test, Adapted for Use in the United States

The USAUDIT [46] is a 10-item measure used to identify hazardous drinking. The scale response is scored from the least frequent, 0 (*never*) to the most frequent, 6 (*daily)*. Scores of ≥8 have been shown to detect hazardous drinking, and a cutoff point <8 has been suggested as a cutoff with higher sensitivity for women [46,47]. The Alcohol Use Disorders Identification Test was found to have a median Cronbach *α* of .83 and a test-retest reliability of 0.92 [47].

#### Short Inventory of Problems-Revised

The Short Inventory of Problems-Revised (SIP-R) [48] assesses alcohol-related problems through 15 questions scored on a 4-point Likert scale from 1 (*never*) to 4 (*daily or almost daily*). Higher scores indicate more life problems related to alcohol use [49]. Among problem drinkers, the SIP-R demonstrated good concurrent validity and internal consistency [49-51].

#### Timeline Followback

The Timeline Followback (TLFB) [52] assesses the quantity and frequency of alcohol consumption. Participants reported the number of drinks they had consumed each day for the past 30 days [52]. When compared with daily interviews using a smartphone, the TLFB showed concurrent validity; however, this diminished over time as participants were less likely to remember the number of drinks they had per day [53].

#### Readiness to Change Questionnaire

The Readiness to Change Questionnaire (RTCQ) [54] is a 12-item questionnaire that measures the stage of change. The scores were calculated to categorize a participant in the “precontemplation,” “contemplation,” or “action” stage of change regarding changing their drinking behavior [54]. The RTCQ demonstrated a test-retest reliability of about 0.80 per scale and a Cronbach *α* of about .80 per scale [54].

#### System Usability Scale

The 10-item System Usability Scale (SUS) [55] measures a user’s experience of a product’s usability. The SUS was found to be reliable, with a Cronbach *α* of .91 [56]. Participants in the app and chatbot groups completed the SUS to assess the usability of both tools.

### Data Collection

Screening and administering consent forms and baseline and follow-up surveys were all done using the web-based survey platform *Qualtrics*. Data were downloaded from *Qualtrics* in CSV format for review and analysis.

#### Screening and Consent

Completed screenings, including the USAUDIT, were automatically scored using *Qualtrics* to determine eligibility. Scores of ≤8 indicated that they did not answer all 9 of the demographic criteria questions required for eligibility (eg, age and residency in the United States); scores of ≥9 were considered eligible. Individuals meeting the demographic criteria were eligible if their USAUDIT score fell between 7 and 24 for females or between 8 and 24 for males. The cutoff criteria were set to reflect the minimum cutoff score for detecting hazardous drinking [57]. Those who scored >24 were not eligible owing to safety concerns; USAUDIT scores of >25 indicate a moderate to severe alcohol use disorder or dependence [57]. These individuals instead received an automated message providing treatment options and mental health resources, including the SAMHSA treatment locator and the national suicide prevention hotline. If they met the eligibility criteria, *Qualtrics* was used to automatically link them to the web-based consent form.

#### Baseline

Baseline survey links were emailed to participants once they were manually reviewed by the study team to confirm their eligibility. Up to 3 reminder emails were sent to complete the baseline survey, if necessary.

#### 12-Week Follow-up

Follow-up surveys were matched to the baseline surveys for each participant. An email with a link to the follow-up survey was manually sent to each participant as they became eligible (ie, after they had used the app or chatbot for 12 weeks, or 12 weeks after their study enrollment for the control group). To encourage a high follow-up response rate, up to 3 reminder emails were sent and up to 3 phone reminder calls were made.

#### Participant Validation

To ensure that the study did not enroll participants who were only seeking renumeration (“phishers”), further participant validation checks were conducted before emailing the baseline survey link, including reviewing the collected data for identical email addresses, multiple duplicate IP addresses, and geolocation data. At follow-up, additional validation checks were conducted. We compared the participants’ demographic responses from baseline to follow-up. In this step, 13 participants reported significant inconsistencies in demographics from baseline to follow-up (eg, they reported inconsistent answers to questions about their ethnicity and gender between baseline and follow-up). We were unable to verify these participants through a telephone call, and they were thus removed from the analyses to ensure valid responses.

#### Sample

**Figure 1:**
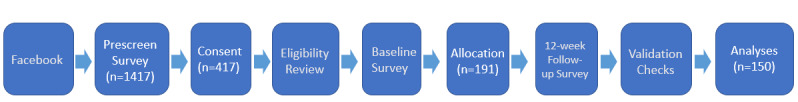
Participant flow.

We recruited 1417 participants in total, including those who completed the prescreen. Of these, 417 patients were eligible and consented to participate. After examining participant responses regarding study eligibility, additional participants were removed because of having numerous prescreen submissions under the same IP address which represented *phishing*, or the automatic scoring through the prescreen allowing ineligible participants into the study (eg, they indicated being currently in alcohol treatment which was an exclusion criteria). Subsequently, the baseline surveys were sent to 197 participants. A few participants (n=6) were further found to be ineligible after examining their prescreening surveys, leaving 191 eligible baseline surveys. At follow-up, 163 participants completed the survey. After removing 13 participants owing to failing our validation checks, we analyzed data from 150 participants, 55 (36.7%) app users, 50 (33.3%) bot users, and 45 (30%) participants in the delay group. Figure 1 shows the flow of this study. All analyses were conducted using SPSS (version 27; IBM Corp).

### Ethics Approval

The study was reviewed and approved by the University of Alaska Anchorage institutional review board (1521800).

## Results

### Sample Characteristics

Table 1 shows demographics from each intervention group.

**Table 1:** Sociodemographic characteristics by intervention group.

Characteristics			App (n=55)		Bot (n=50)		Delay (n=45)
Age (years), mean (SD)			42.58 (13.49)		40.82 (12.54)		41.51 (13.07)
**Sex, n (%)**							
	Male	25 (46)		22 (44)		16 (36)	
	Female	30 (54)		28 (56)		29 (64)	
**Race and ethnicity, n (%)**							
	African American	5 (9)		8 (16)		3 (7)	
	White	44 (80)		36 (72)		37 (82)	
	Asian American	2 (4)		0 (0)		5 (11)	
	Alaska Native or American Indian	0 (0)		1 (2)		0 (0)	
	Hispanic or Latinx	3 (6)		4 (8)		0 (0)	
	Other	1 (2)		1 (2)		0 (0)	

### Change in Drinking Measure From Baseline to Follow-up

**Figure 2:**
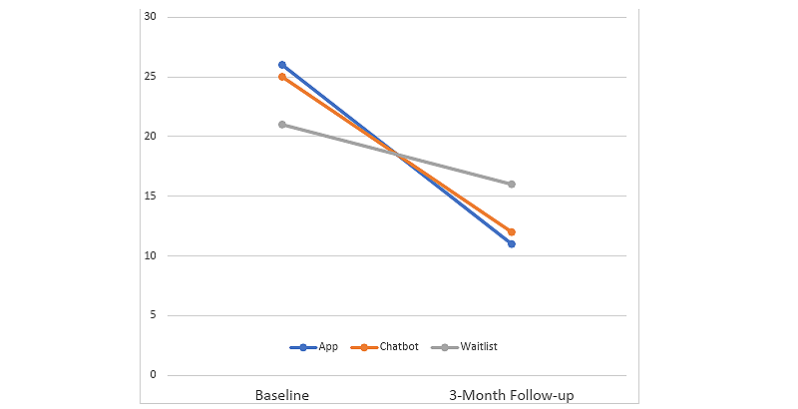
Percentage of days with hazardous drinking from baseline to follow-up.

Missing data were imputed using multiple imputation. TLFB data correlated with age, gender, and SIP-R data. Multiple imputation was performed using age, gender, SIP-R, and TLFB data as predictor variables for missing TLFB data with 5 data sets imputed. A total of 36 respondents were excluded from the TLFB analyses owing to insufficient TLFB data, resulting in a total of 114 participants from the app (n=42, 36.8%), bot (n=39, 34.2%), and delay (n=33, 29%) groups for the TLFB analyses. These participants did not complete any of the 30-day TLFB survey questions at either baseline or follow-up; therefore, multiple imputation was not possible with these participants. We calculated drinks per day (DPD), percent days abstinent (PDA), and heavy drinking days (HDD). Table 2 shows the descriptive data for each intervention group regarding drinking variables at baseline and follow-up. Figure 2 shows the percentage of days spent drinking hazardously (our primary outcome variable) from baseline to follow-up among the 3 groups.

**Table 2:** Means and SDs of measures by intervention group.

Measure			App					Bot					Delay		
			Baseline		Follow-up		Baseline			Follow-up		Baseline			Follow-up
**Timeline followback measures (N=114), n (%)**			42 (36.8)		42 (36.8)		39 (34.2)			39 (34.2)		33 (28.9)			33 (28.9)
	DPD,^a^ mean (SD)	2.69 (1.72)		1.51 (1.04)		2.64 (1.26)			1.75 (1.17)		2.50 (1.14)			1.88 (1.41)	
	PDA^b^ (%), mean (SD)	24 (28)		44 (31)		22 (22)			36 (29)		21 (24)			39 (32)	
	HDD,^c^ mean (SD)	7.69 (8.33)		3.29 (5.50)		7.49 (7.98)			3.54 (6.76)		6.30 (5.65)			4.94 (6.92)	
**Drinking outcome measures (N=150), n (%)**			55 (36.7)		55 (36.7)		50 (33.3)			50 (33.3)		45 (30)			45 (30)
	AUDIT,^d^ mean (SD)	15.25 (4.35)		12.62 (7.24)		16.28 (3.49)			14.76 (7.01)		15.60 (4.42)			14.62 (7.50)	
	RTCQ,^e^ mean (SD)	2.35 (0.55)		2.40 (0.68)		2.14 (0.50)			2.62 (0.57)		2.35 (0.57)			2.35 (0.65)	
	SIP-R,^f^ mean (SD)	11.69 (7.25)		8.16 (6.80)		12.68 (10.28)			9.86 (7.95)		13.51 (9.46)			9.53 (8.45)	

^a^DPD: drinks per day.

^b^PDA: percent days abstinent.

^c^HDD: heavy drinking days (as defined by ≥4 DPD for females and ≥5 DPD for males).

^d^AUDIT: Alcohol Use Disorder Identification Test, Adapted for Use in the United States.

^e^RTCQ: Readiness to Change Questionnaire.

^f^SIP-R: Short Inventory of Problems-Revised.

### Alcohol Consumption Results by Group

Repeated measures ANOVA were conducted on the 3 dependent drinking variables calculated from the TLFB, DPD, PDA, and HDD. The within-subjects variable was time (baseline to follow-up), and the between-subjects variable was the intervention group (app, bot, or delay). There was a significant effect of time on DPD (*F*_2,111_=55.93; *P*<.001; *η*^2^=0.34), PDA (*F*_2,111_=42.00; *P*<.001; *η*^2^=0.27), and HDD (*F*_2,111_=28.18; *P*<.001; *η*^2^=0.20), and all were large effect sizes. There was no significant interaction between time and group for both DPD (*F*_2,111_=1.74; *P*=.18; *η*^2^=0.03) and HDD (*F*_2,111_=2.27; *P*=.11; *η*^2^=0.04). These results indicate that all participants reduced their drinking significantly between baseline and follow-up, and there was no significant difference in the reduction in drinking based on the group they were randomized to.

Repeated measures ANOVA were conducted on three dependent drinking-related variables: AUDIT, RTCQ, and SIP-R. The within-subjects variable was time (baseline to follow-up), and the between-subjects variable was the intervention group (app, bot, or delay). There was a significant effect of time on the SIP (*F*_2,147_=24.76; *P*<.001; *η*^2^=0.14), with a large effect size. There was a significant effect of time with a medium effect size on AUDIT (*F*_2,147_=10.97; *P*=.001; *η*^2^=0.07) and RTCQ (*F*_2,147_=7.79; *P*=.006; *η*^2^=0.05). Between- and within-subjects ANOVAs were conducted to compare the intervention groups for the 3 dependent drinking-related variables. There was no significant interaction between time and group for AUDIT or SIP-R scores, indicating that all 3 intervention groups significantly improved on these drinking measures between baseline and follow-up but did not differ from one another. There was a significant interaction between time and group for the RTCQ (*F*_2,147_=5.62; *P*=.004; *η*^2^=0.07) with a medium effect size, indicating that readiness to change scores varied by intervention group, with those in the bot group improving significantly more between baseline and follow-up than those in the app or delay group. The intervention group did not effect change in SIP or AUDIT scores for participants.

### App Versus Bot Utilization Results

Three different variables were calculated to measure utilization. We calculated from the Step Away database that the app and the bot collected, the number of times users clicked on a function of the app as the total number of visits they made to each of the interventions. Duration of use was calculated as the time between the date the user downloaded the app and their date of last use. Active days used was calculated as the number of days that a user was actively using the app, which was defined as having entered the app and clicked on at least one feature. Utilization data from 18 bot users were missing, indicating that these participants either did not use the bot at all or did not enter their personal identification number at the start of the study, which was required to match their utilization data to their study ID. Therefore, the results must be interpreted with caution and the means for the bot utilization variables are likely lower than those presented here. Independent sample 2-tailed *t* tests were conducted between the means of each variable to determine whether there were significant differences between app and bot users. Duration of use significantly differed between app users and bot users (*F*_85_=12.23; *t*_85_=2.45; *P*=.02), with duration of use being higher among app users (mean 50.71, SD 49.02) than among bot users (mean 27.16, SD 30.54). Total visits were higher for app users (mean 33.96, SD 38.16) than for bot users (mean 24.56, SD 28.68; *F*_85_=1.53; *t*_85_=1.21; *P*=.22), and the average active days used was higher for app users (mean 22.62, SD 29.08) than bot users (mean 19.63, SD 26.30; *F*_85_=.02; *t*_85_=.48; *P*=.88), although neither of these differences were statistically significant.

### Effect of Utilization of the Intervention on Change in Drinking Variables

Significant direct effects were shown for the duration of use of the app or bot on the change in average DPD and PDA. Increased duration of use predicted a greater decrease in DPD (*β*=.01, SE 0.00; *β*=.25, 95% CI 0.00-0.01; *P*=.04), and increased duration of use predicted a greater change in the increase of PDA (*β*=−.18, SE 0.07; *β*=−.29, 95% CI −0.32 to −0.03; *P*=.02). There were trends of utilization increasing change in drinking that were not significant, including an effect of total visits on the change in PDA (*β*=−.16, SE 0.10; *β*=−.20, 95% CI −0.35 to 0.03; *P*=.10), of active days of use on the change in PDA (*β*=−.21, SE 0.12; *β*=−.21, 95% CI −0.45 to 0.03; *P*=.08), and of duration of use on the change in HDD (*β*=.03, SE 0.02; *β*=.23, 95% CI −0.00 to 0.07; *P*=.06). These trends indicated that increased use of the app or bot led to greater decreases in drinking and increases in abstinence.

### Usability Ratings for the App Versus Bot

Means from the overall SUS score were compared between app and bot users using independent sample 2-tailed *t* tests. App users rated a higher mean SUS score (mean 66.35, SD 19.68) than bot users (mean 61.70, SD 25.40), but this difference was not statistically significant. A typical acceptable SUS score is approximately 70, and scores <50 are considered unacceptable [56]. Table 3 presents the mean (SD) for the app and bot participants for each SUS question.

**Table 3:** System Usability Scale by intervention group.

Items	App (n=52), mean (SD)	Bot (n=48), mean (SD)
1. I think I would use the Step Away app/chatbot frequently.	3.33 (1.31)	3.15 (1.41)
2. I found the Step Away app/chatbot unnecessarily complex.	2.46 (1.21)	2.65 (1.44)
3. I thought the Step Away app/chatbot was easy to use.	3.83 (1.20)	3.56 (1.41)
4. I think that I would need assistance to be able to use the Step Away app/chatbot.	1.92 (1.25)	2.19 (1.35)
5. I found the various functions in the Step Away app/chatbot well-integrated.	3.42 (1.18)	3.35 (1.42)
6. I thought there was too much inconsistency in the Step Away app/chatbot.	2.35 (1.06)	2.85 (1.38)
7. I would imagine that most people would learn to use the Step Away app/chatbot very quickly.	3.94 (0.94)	3.88 (1.20)
8. I found the Step Away app/chatbot very cumbersome to use.	2.67 (1.31)	2.94 (1.48)
9. I felt very confident using the Step Away app/chatbot.	3.75 (1.05)	3.75 (1.25)
Total score	66.35 (19.68)	61.70 (25.40)

## Discussion

### Principal Findings

This study sought to develop a chatbot version of the empirically supported app, Step Away, and conduct a pilot trial to determine if a chatbot version could provide enhanced use and outcome effectiveness over the app version. We also sought to pilot trial a methodology that included randomly assigning participants who were hazardously consuming alcohol and interested in making a change to their drinking to one of three conditions: the Step Away app, the Step Away bot, and a waitlist control condition.

Results from this pilot study indicated that self-reported alcohol consumption from baseline to the 12-week follow-up decreased substantially in all groups. Effect sizes suggested that changes in alcohol consumption and drinking-related problems were within the large effect range. However, the results also suggested that there were no statistically significant differences in alcohol consumption variables between the 3 groups, suggesting that the waitlist control condition changed their drinking at a similar level to the intervention groups. The lack of statistical significance could potentially be related to an inadequate sample size (the purpose of the study was not to provide a robust test of effectiveness and power calculations were not performed). It is also likely that the waitlist control condition, owing to their expressed desire to change their drinking, had a strong (and positive) reaction to being assessed at baseline for their alcohol consumption and drinking-related variables, such as life problems stemming from alcohol consumption [58]. A delayed assessment of the control group (until the intervention groups are assessed at follow-up) is a highly recommended strategy for managing this possibility.

Another focus of this study, which is perhaps equally important for alcohol outcome analysis, is the utilization assessment of the 2 interventions. In contrast to our expectations, our results suggested that the app was used more frequently and for a longer time than the bot. There are 2 possible explanations for this observation. The Step Away app was originally developed in 2013 and has undergone 4 major revisions based on user input and recommendations. A finding that has emerged frequently from research on Step Away is that the app’s ability to provide self-monitoring and tailored feedback to the user is a key driver of utilization [43,59,60], which has resulted in the feedback feature being prominent and well-developed. This was the first version of the bot, and, like many first versions, it had some unforeseen limitations. First, similar to all Facebook Messenger bots, it provided a daily SMS text message prompting the user to check in with the bot; however, if they did not respond, the bot did not continue to provide prompts. It relied on the user to return to it on subsequent days to re-engage with it. We heard from some users that they thought the bot disengaged from them as it stopped providing reminders to complete their daily interviews. This notification problem did not exist with the app and app users were notified daily regardless of their response to the prompts.

Another factor related to the feedback is that although an app can provide sophisticated graphs and other pictographic representations of progress, bots are much more limited in this regard (a limitation that existed when we developed the bot). The bot’s feedback, although similar in content to the app, was more simplistic and perhaps not captivating. Finally, on 2 occasions the bot *crashed* (ie, ceased to function) during the study, possibly owing to complications related to its dependence on Facebook Messenger, and making it difficult for our developers to resolve promptly. The app also crashed once during the study, but the problem was immediately corrected.

Although this study indicated numerous benefits of the app over the bot, one finding favored the bot. Bot participants were found to have a greater change in motivation to change their drinking compared with the app. It is possible that the conversational tone and the feeling that the bot was more like talking to a person could have enhanced users’ motivation to change their drinking habits. Future research should explore this possibility.

### Limitations and Future Directions

This study had numerous limitations, many because of its pilot nature, which are related to recommendations for future research. First, the sample was insufficient for detecting small to medium effect sizes, and our findings reflected this limitation. We estimated that a *sample size* of 195 would provide enough power to detect between-group effect sizes, as shown in this study. Second, the methodology for assessing the waitlist control condition likely resulted in reactivity to assessment phenomena, which made detecting differences between the interventions and the control condition challenging. Future studies in this area would be wise, if using a waitlist control condition, to delay assessment, a method that has been used successfully in other studies [14]. We also had limited time to undertake this study and our 12-week follow-up period may have been insufficient for differences between interventions to emerge. Previous research with Step Away showed that participants continued to reduce their alcohol intake at 6 months [43] and that 45% of participants were still actively engaged at the 6-month follow-up. A 12-month follow-up would provide a more detailed picture of how users remain engaged with the interventions over time and how this engagement is related to improvement. Finally, although this study set out to contrast the 2 interventions and determine which had higher use and effectiveness, this contrast is perhaps not ideal. A more beneficial strategy may involve combining these technologies. Throughout the development of the bot, we realized that both bots and apps each have their unique strengths. Apps can provide highly captivating images and graphs depicting progress, while bots provide the feeling that the user is interacting with someone or something, which has the potential to increase motivation and overall engagement. Apps are also more self-guided than bots and allow the user to interact in a more self-directed, timely manner than a bot, which guides the user by suggesting modules and responding in a conversational manner. It is possible to merge these 2 technologies into 1 system by placing a bot within an app. Thus, it may be that the *best in class* smartphone-based system for reducing drinking is a hybrid of a bot and an app, a promising direction for future interventions.

### Conclusions

Research on smartphone-based interventions for alcohol problems has been promising, but more research into factors related to use and long-term effectiveness is urgently needed. This study indicated that the app and bot interventions were related to substantial improvements in drinking, as was the waitlist control condition, which resulted in inconclusive results. We found that the app was more highly used, which may have been related to some bot limitations that are easily addressable, such as improved prompting over time and greater system stability, which has undoubtedly improved since the development of the Step Away bot. Future research should use longer follow-ups, more sophisticated methodology regarding the timing of the assessment for the control condition, and perhaps leveraging the 2 technologies to develop a hybrid system that has the beneficial elements of an app and a bot.

## Appendix

Multimedia Appendix 1 CONSORT-EHEALTH checklist (V 1.6.1).

## Abbreviations

DPD: drinks per day
HDD: heavy drinking days
PDA: percent days abstinent
RTCQ: Readiness to Change Questionnaire
SIP-R: Short Inventory of Problems-Revised
SUS: System Usability Scale
TLFB: Timeline Followback
USAUDIT: Alcohol Use Disorders Identification Test, Adapted for Use in the United States
